# Chronic Overlapping Pain and Central Sensitization in Chronic Pelvic Pain: Do Positive and Negative Affect Play a Role?

**DOI:** 10.1155/prm/9124509

**Published:** 2026-05-27

**Authors:** Shreela Palit, Meryl J. Alappattu, Jenna M. Wilson, Jessica S. Heft, Emily J. Bartley

**Affiliations:** ^1^ Center for Healthcare Delivery Science, Nemours Children’s Health, Jacksonville, Florida, USA, nemours.org; ^2^ College of Public Health and Health Professions, University of Florida, Gainesville, Florida, USA, ufl.edu; ^3^ Anesthesiology Perioperative and Pain Medicine, Brigham and Women’s Hospital, Boston, Massachusetts, USA, brighamandwomens.org; ^4^ College of Medicine, University of Florida, Gainesville, Florida, USA, ufl.edu; ^5^ College of Dentistry, University of Florida, Gainesville, Florida, USA, ufl.edu

**Keywords:** central sensitization, chronic overlapping pain conditions, COPCs, pelvic pain, positive affect, resilience

## Abstract

Chronic pelvic pain (CPP) has a complex etiology and a high degree of coprevalence with other chronic overlapping pain conditions (COPCs). Evidence suggests that central sensitization contributes to these conditions; however, the psychological risk and resilience factors influencing the relationship between COPCs and central sensitization have received limited attention. Therefore, the aim of this study was to examine whether positive and negative affect moderate the relationship between COPCs and central sensitization in individuals with CPP. Participants (*N* = 197) completed the Central Sensitization Inventory and Positive and Negative Affect Schedule and self‐reported the presence of diagnosed COPCs. Results indicated that greater symptoms of central sensitization were associated with a greater number of COPCs (*p* < 0.01). Higher levels of positive affect were related to lower central sensitization (*p* < 0.01) and fewer COPCs (*p* < 0.05), while higher negative affect was associated with greater central sensitization (*p* < 0.01). After controlling for pelvic pain duration, a greater number of COPCs were associated with greater central sensitization, but only among individuals with lower positive affect (*p* = 0.01). No significant moderation effects were observed for negative affect (*p* = 0.13). The findings from this cross‐sectional study suggest that lower positive affect may have an adverse effect on central sensitization in those with a greater number of COPCs. Individuals with CPP, especially those experiencing multiple chronic pain conditions, may benefit from interventions aimed at increasing positive affect.

## 1. Introduction

Chronic pelvic pain (CPP) is characterized by pain localized below the umbilical/lower abdominal region and between the hips that persists for 3 months or longer. CPP leads to significant functional and psychosocial impairment and affects upward of 25% of people globally [1–4]. Though predominantly affecting women, CPP is not exclusive to this group, with between 2% and 16% of men affected by the presence of pelvic pain (e.g., chronic prostatitis/CPP syndrome) [5]. CPP has a complex etiology and heterogeneous presentation, and can arise from one or more underlying causes, many of which overlap [2, 6].

CPP often co‐occurs with other pain disorders collectively referred to as chronic overlapping pain conditions (COPCs). COPCs are characterized by a group of 10 frequently comorbid chronic pain disorders (i.e., temporomandibular disorder, fibromyalgia, irritable bowel syndrome, vulvodynia, myalgic encephalomyelitis/chronic fatigue syndrome, interstitial cystitis/painful bladder syndrome, endometriosis, chronic tension‐type headache, migraine headache, and chronic low back pain [7]) that share common pathophysiological mechanisms, risk factors (e.g., early life adversity and female sex), and psychosocial correlates, such as depression and anxiety. Importantly, COPCs represent a heterogeneous grouping of conditions with variability in diagnostic criteria and methods of assessment across studies and are often identified via self‐report or symptom‐based screening rather than standardized diagnostic procedures. This variability may ultimately contribute to differences in prevalence estimates and observed associations across studies.

Despite conceptual overlap, research has traditionally examined COPCs in isolation. As a result, patients with overlapping pain conditions have been underrepresented in the literature, limiting the generalizability of findings and obscuring shared mechanisms across disorders. Further, routine screening of COPCs is not standard practice, and the lack of standardized screening instruments has hindered systematic identification of these conditions in both clinical and research settings.

Clinically, the co‐occurrence of multiple pain conditions is associated with poorer pain‐related outcomes. Compared to individuals without multiple chronic pain conditions, those with COPCs experience greater pain severity, lower psychosocial functioning (e.g., disrupted mood and disturbed sleep), and poorer response to typical interventions [2, 6]. Consistent with this, a recent investigation demonstrated that a higher number of COPCs were associated with greater pelvic pain severity, pain interference, and pain impact in women with and without endometriosis [8].

CPP and COPCs impact individuals across sex/gender, socioeconomic status, and race/ethnicity, though prevalence may vary (e.g., these conditions disproportionately impact more women than men [7]). While numerous mechanisms underlie the development and maintenance of CPP and other COPCs across individuals, central sensitization (CS) is a primary shared contributory factor, which refers to heightened pain that results from changes (e.g., increased sensitivity of neurons) within the central nervous system [9]. CS contributes to problems with mood, sleep, cognitive functioning, and fatigue, as well as greater sensitivity to painful and nonpainful stimuli in individuals with chronic pain, including CPP [10–12]. Understanding the role that CS plays in CPP is crucial, given that traditional treatments often involve peripherally focused interventions, such as surgical procedures that target the sensory dimension of pain [7, 13]. These conservative biomedical approaches also fail to consider the psychological contributors, which regulate the pain experience and influence the pain–CS relationship. Moreover, traditional treatments are unlikely to alleviate pain or halt disease progression when the underlying mechanisms are more centrally acting [10, 12, 14]. As with most chronic pain conditions, the presence of COPCs necessitates a multimodal treatment approach that extends beyond medical and surgical interventions [2] in order to address the psychosocial factors that accompany pain.

Existing studies have primarily focused on the impact of risk and vulnerability factors, including psychosocial variables, on CPP. Negative affect (e.g., depression and anxiety) frequently accompanies CPP, with higher rates of psychological disorders found in individuals with CPP, compared to those without [15]. Additionally, negative affect has been linked to increased pain severity and heightened CS [16, 17], and individuals with COPCs often report greater levels of negative affect than those without COPCs [18]. However, there is limited research exploring how risk factors, such as negative affect, may interact with and influence the relationship between COPCs and CS in this population. It is possible that negative affect may amplify the association between elevated CS and a higher number of COPCs.

More recently, studies have begun to explore the role of protective factors (e.g., resilience and positive affect) that mitigate adverse pain outcomes. For example, our previous work demonstrated that pain resilience moderated the relationship between negative pain beliefs and movement‐evoked pain in older adults with chronic low back pain [19]. Similarly, a recent study found that individuals with urological CPP exhibited lower levels of general resilience, particularly among those who also reported widespread pain or COPCs [20]. Positive emotional processes may play an important role in this context, as positive emotions have been consistently linked to lower pain severity among both pain‐free individuals and those with chronic pain [21–24]. This pattern aligns with the Broaden‐and‐Build theory of positive emotions [25], which signifies that positive affective states foster adaptive cognitions and approach‐oriented behaviors, thereby facilitating the cultivation of personal resources that optimize physical and psychological functioning. Notably, individuals with COPCs often report lower levels of positive affect than those without COPCs [18], suggesting that lower positive affect may reflect reduced protective capacity, whereas higher positive affect may attenuate the impact of elevated CS on the number of COPCs. Understanding whether positive affect attenuates, and negative affect amplifies, the relationship between CS and COPCs may help identify targets that promote resilience or exacerbate risk.

Building on this work, a growing body of evidence suggests that positive affect may mitigate the adverse cognitive and emotional consequences of chronic pain and, in some cases, exert a stronger influence on the pain experience compared to negative affect [23]. In an early study, individuals with CPP and high negative affectivity who engaged in expressive writing about pelvic pain–related stress showed increases in positive affect [26]. More recently, van Barneveld and colleagues used ecological momentary assessment to investigate positive and negative affect in women with and without endometriosis. Their findings revealed that greater abdominal pain in women with endometriosis was associated with higher negative affect and lower positive affect compared to healthy controls [27]. These results reinforce the notion that positive and negative affect are distinct emotional constructs that independently shape the pain experience, signifying the importance of both as complementary strategies to reduce symptom burden in CPP. Further, a systematic review and meta‐analysis in adults with chronic pain found an inverse relationship between positive affect and pain severity, supporting the potential benefit of incorporating positive affect–enhancing strategies within psychological pain management treatments [28].

Gaining a better understanding of the role of protective and vulnerability factors that may contribute to mechanisms underlying CPP can inform and improve ways to tailor CPP treatment approaches. This study aimed to examine 1) the associations between positive affect and negative affect with CS and COPCs, 2) the relationship between CS and COPCs, and 3) to identify whether positive and negative affect moderate the relationship between COPCs and CS in individuals with CPP. We hypothesized that 1) positive affect would be negatively associated with CS and COPCs, while negative affect would be positively associated with these outcomes, and 2) CS and COPCs would be positively related (i.e., greater CS would be associated with increased presence of overlapping pain conditions). Consistent with previous work [19, 29] demonstrating that lower levels of resilience factors, such as positive affect, are associated with adverse pain outcomes, we further hypothesized that positive affect would moderate the association between CS and COPCs, such that this relationship would be stronger at lower levels of positive affect.

## 2. Materials and Methods

### 2.1. Procedures

Study data were collected and managed using REDCap (Research Electronic Data Capture), a secure, web‐based application designed to support data capture for research studies. Participants recruited through REDCap were informed about the study via advocacy groups, social media (e.g., Facebook and Twitter), flyers, newspaper advertisements, and local gynecology clinics. Interested individuals accessed a study link directing them to the REDCap survey, where they first completed informed consent followed by screening items to confirm eligibility. If eligible, participants then proceeded to the full survey. As a secondary recruitment source, participants were also recruited through Qualtrics, an online survey platform that provides access to national samples through established research panels. Potential participants within the Qualtrics panel were invited to complete the survey and were similarly screened for eligibility before participation.

The initial screening included questions regarding CPP, age, biological sex, and country of origin. Individuals meeting eligibility criteria were asked to proceed with the remaining survey questionnaires. To be included in the study, participants had to be ≥ 18 years of age, reside in the United States, and endorse pain in the pelvic region with pain occurring for the past 3 months, consistent with common definitions of CPP [30]. Individuals reporting pelvic pain of less than 3‐month duration were excluded to ensure the sample reflected chronic pain rather than acute pain. Adults were targeted because prevalence estimates of CS and COPCs are better characterized in adult populations, and the measures used in this study have largely been validated in adults. Additionally, the sample was restricted to U.S. residents to maintain consistency in the healthcare context and the language of study measures.

Data were checked for validity and reviewed by four members of the research team to ensure attention and accuracy of responding. After completion of the survey, participants were compensated $10. All procedures were approved by the University of Florida Institutional Review Board (IRB#202001838) and participants provided electronic informed consent before study procedures.

### 2.2. Measures

#### 2.2.1. COPCs

Participants completed a self‐report medical history form in which they were presented with a list of conditions and asked: “Has a health care professional told you that you currently have any of the following health conditions?” Participants indicated whether they had been diagnosed with the following COPCs: 1) vulvodynia, 2) irritable bowel syndrome, 3) temporomandibular disorder, 4) myalgic encephalomyelitis/chronic fatigue syndrome, 5) interstitial cystitis/painful bladder syndrome, 6) fibromyalgia, 7) endometriosis, 8) chronic tension‐type or migraine headache, and 9) chronic low back pain [7]. To capture the cumulative burden of COPCs, and aligning with prior research, a single summary score was created by counting the number of COPCs reported [8, 18, 31].

#### 2.2.2. Central Sensitization Inventory (CSI)

The CSI is a widely used self‐report measure of CS [32, 33]. It was administered to identify the presence of central pain mechanisms and assess the frequency of 25 health symptoms (e.g., “My muscles feel stiff and achy”) related to CS or central sensitivity syndromes [34]. Items are rated from 0 (*never*) to 4 (*always*) for a total possible score of 100. Continuous scores obtained from the CSI were used in analyses. In this sample, the Cronbach’s alpha for the CSI was 0.91.

#### 2.2.3. Positive and Negative Affect Schedule (PANAS)

Positive affect and negative affect were assessed using the trait version of the PANAS [35]. Respondents were presented with a 20‐item scale consisting of 10 positively valenced and 10 negatively valenced terms and were asked to indicate the extent to which they generally feel. Each term is rated on a 5‐point scale ranging from 1 (*very slightly or not at all*) to 5 (*extremely*) resulting in scale scores for positive and negative affect. Higher scale scores correspond to increased positive and negative affect. Internal consistency estimates were found to be high for both subscales in the current sample: α = 0.88 for positive affect and 0.91 for negative affect.

### 2.3. Statistical Analyses

All analyses were performed using SPSS 27, and the significance level was set at *p* ≤ 0.05 (2‐tailed). Descriptive statistics were used to calculate means, standard deviations, and frequencies for demographic variables. Relationships between demographic characteristics and variables of interest were examined via bivariate correlations. An empirical approach was used to select covariates for moderation analyses, such that demographic variables significantly associated with both the predictor (number of COPCs) and outcome (CS) were included as covariates to account for potential confounding while maintaining model parsimony. Two moderation analyses were conducted using Hayes PROCESS macro to examine whether positive and/or negative affect moderated the relationship between the number of COPCs and CS. Before conducting moderation analyses, assumptions of linear regression for the association between COPCs and CS were evaluated through visual inspection of scatterplots with fitted regression and LOESS smoothing lines, examination of residual plots, and testing of a quadratic term for COPCs. These diagnostics indicated no meaningful departures from linearity. Continuous predictors were mean‐centered to improve interpretability and reduce multicollinearity in interaction terms. The PROCESS macro uses regression‐based path‐analytic modeling and automatically mean center variables and provides conditional effects for moderation models.

## 3. Results

### 3.1. Participant Characteristics

Table 1 presents demographic variables (means, *SD*s, and counts). Of the 345 participants screened, 197 met study inclusion criteria and were included in analyses. Participants were excluded due to the following: not meeting inclusion criteria (*n* = 50, 14.4%), pelvic pain diagnosis not reported (*n* = 6, 1.7%), partial completion of primary study measures (*n* = 33, 9.6%), current pregnancy (*n* = 2, 0.6%), and invalid responding (*n* = 37, 10.7%). Participants were predominantly female (78%), white/Caucasian (79%), had a college degree (62%), and were not married (59%). The average age was 36 years (range: 18–72 years). The average pelvic pain duration reported was 7.8 years (range: 3 months to 45 years). The reported type(s) of causes for pelvic pain conditions are displayed in Table 2. The most endorsed pelvic pain conditions included CPP, endometriosis, and dysmenorrhea. The following COPCs were reported: endometriosis (*n* = 74, 37.6%), chronic low back pain (*n* = 36, 18.3%), chronic tension/migraine headache (*n* = 33, 16.8%), irritable bowel syndrome (*n* = 27, 13.7%), fibromyalgia (*n* = 21, 10.7%), interstitial cystitis/painful bladder syndrome (*n* = 18, 9.1%), vulvodynia (*n* = 8, 4.1%), myalgic encephalomyelitis/chronic fatigue syndrome (*n* = 8, 4.1%), and temporomandibular disorder (*n* = 7, 3.6%).

**TABLE 1:** Demographics.

Characteristic	*M* (or *N*)	*SD* (or %)
Age (years)	35.6	11.2
Sex assigned at birth		
Female	153	77.7
Male	44	22.3
Gender identity		
Woman	152	77.2
Man	43	21.8
Nonbinary	1	0.5
Self‐described	1	0.5
Race		
Asian	3	1.5
Black	26	13.2
White	156	79.2
American Indian	1	0.5
Other or multiple races	11	5.6
Education		
No HS diploma	5	2.5
HS graduate/GED	26	13.2
Some college	38	19.3
Occupational tech program	5	2.5
Two‐year degree	27	13.7
Four‐year degree	58	29.4
Master’s degree	25	12.7
Professional school degree	5	2.5
Doctoral degree	8	4.1
Marital status		
Married	81	41.1
Single	73	37.1
Cohabitating	20	10.2
Widowed	3	1.5
Divorced	16	8.1
Separated	4	2
Income		
≤$9999	10	5.1
$10,000–24,999	20	10.2
$25,000–49,999	60	30.5
$50,000–74,999	39	19.8
$75,000–99,999	26	13.2
$100,000–149,000	27	13.7
≥$150,000	10	5.1
Prefer not to answer	5	2.5
Pelvic pain duration (months)	93.9	108.1
Chronic overlapping pain conditions		
0	67	34
1	67	34
2	39	19.8
≥ 3	24	12.2

*Note:* HS = high school; GED = general education development.

**TABLE 2:** Self‐reported cause of pelvic pain.

Condition	N	Percentage
Chronic pelvic pain	66	33.5
Endometriosis^∗^	74	37.6
Dysmenorrhea	40	20.3
Vulvodynia^∗^	16	8.1
Fibroids	18	9.1
Prostatitis	3	1.5
Interstitial cystitis^∗^	15	7.6
Pelvic inflammatory disease	10	5.1
Levator syndrome	6	3.0
Pelvic congestion syndrome	2	1.0
Rheumatoid arthritis	9	4.6
Osteoarthritis (OA)	8	4.1
OA or joint	13	6.6
Fibromyalgia^∗^	13	6.6
Other	43	21.8
Unknown	17	8.6
Crohn’s disease	2	1.0
Injury	5	2.5
PCOS	2	1.0
Surgery	3	1.5
Kidney stones	1	0.5
Other medical reasons	12	6.1

*Note:* ^∗^indicates conditions that are COPCs; PCOS = polycystic ovary syndrome.

### 3.2. Zero‐Order Correlations

Bivariate correlations between key variables are reported in Table 3. Greater CS was associated with a greater number of COPCs (*p* < 0.01). Higher positive affect was correlated with lower CS and fewer COPCs (*p*’s < 0.01), while higher negative affect was associated with greater CS (*p* < 0.01). Negative affect was not significantly correlated with COPCs (*p* = 0.14). Covariates were selected based on theoretical relevance and their associations with the primary study variables. Among demographic variables examined (sex, income, education, and pelvic pain duration), only pelvic pain duration was significantly associated with both COPCs and CS. Therefore, this variable was included as a covariate in the moderation analyses to account for potential confounding.

**TABLE 3:** Bivariate correlations.

	1	2	3	4	5	6	7	8	9	10	11	12
1. CSI	—											
2. COPCs	0.28^∗∗^	—										
3. Positive affect	−0.30^∗∗^	−0.20^∗∗^	—									
4. Negative affect	0.66^∗∗^	0.14	−0.27^∗∗^	—								
5. Sex	0.04	0.25^∗∗^	−0.08	0.02	—							
6. Age	0.01	−0.001	0.05	−0.09	−0.08	—						
7. Race	−0.08	−0.16^∗^	0.10	0.07	−0.03	−0.10	—					
8. Ethnicity	−0.13	0.03	−0.11	−0.10	−0.02	0.06	−0.14	—				
9. Marital status	0.11	−0.12	−0.11	0.12	−0.15^∗^	−0.20^∗∗^	0.10	−0.12	—			
10. Education	−0.09	0.29^∗∗^	0.01	−0.10	0.17^∗^	0.06	−0.02	−0.07	−0.16^∗^	—		
11. Income	−0.10	0.14^∗^	0.16^∗^	−0.20^∗∗^	0.04	0.05	−0.10	−0.07	−0.32^∗∗^	0.24^∗∗^	—	
12. Pelvic pain duration	0.23^∗∗^	0.36^∗∗^	−0.18^∗^	0.15^∗^	0.28^∗∗^	0.18^∗^	−0.16^∗^	0.06	−0.12	0.17^∗^	0.12	—

*Note:* CSI = central sensitization inventory; COPCs = chronic overlapping pain conditions.

^∗^Correlation is significant at the 0.05 level (2‐tailed).

^∗∗^Correlation is significant at the 0.01 level (2‐tailed).

### 3.3. Exploratory Analysis

Given evidence that the presence of a greater number of COPCs is associated with worse pain‐related outcomes [8, 36], we conducted a Welch’s ANOVA followed by Games–Howell post hoc tests to explore whether there was a stepwise/incremental relationship in average CS as a function of number of COPCs reported. This model indicated significant differences between group means (F [3, 72.79] = 6.16, *p* = 0.001), such that as the number of COPCs increased, average CS increased. However, these differences were only statistically significant when comparing individuals reporting 3 or more COPCs with those reporting 0, 1, or 2 COPCs (*p*’s < 0.05). No other between‐group differences were significant (e.g., between 0 and 1 COPCs).

### 3.4. Moderation Analyses

**FIGURE 1:**
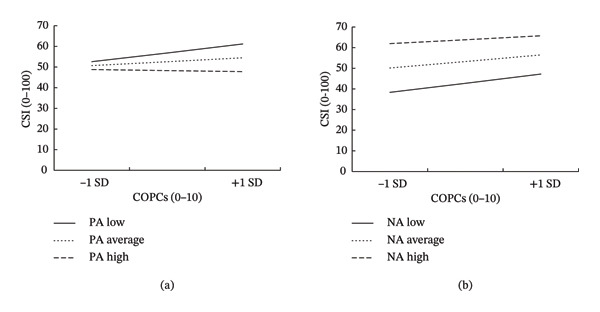
Moderation analyses. *Note.* Relationships between COPCs and CS across low (−1 *SD*), mean, and high (+1 *SD*) levels of (a) PA and (b) NA. A greater number of COPCs were associated with higher CS, but only among those with low PA. NA did not significantly moderate the COPC x CS relationship. COPCs = chronic overlapping pain conditions; CSI = central sensitization inventory; PA = positive affect; NA = negative affect.

Figure 1 presents the results from moderation analyses examining the relationships between 1) COPCs, positive affect, and CS and 2) COPCs, negative affect, and CS. After controlling for pelvic pain duration, the overall model for CS with positive affect as a moderator significantly accounted for 17% of the variance in CS (*F* = 9.19, *p* < 0.001). There was a significant interaction between COPCs and positive affect (ΔR^2^ = 0.02, *p* < 0.05). The conditional effect of COPCs was tested at three levels of positive affect: one standard deviation above the mean, at the mean, and one standard deviation below the mean. Simple slopes analyses revealed that a greater number of COPCs were associated with higher CS only among individuals with low levels of positive affect (*β* = 3.66, *p* = 0.002, 95% CI [1.22, 5.76]), but not among those with average (*β* = 1.61, *p* = 0.17, 95% CI [−0.67, 3.91]) or high (*β* = −0.44, *p* = 0.80, 95% CI [−3.99, 2.95]) levels of positive affect. Thus, this effect was found to be significant only for low positive affect. In contrast, the overall model with negative affect as a moderator significantly accounted for 48% of the variance in CS (*F* = 40.50, *p* < 0.001) after controlling for pelvic pain duration. However, no significant moderation effects were observed for negative affect (*p* = 0.13).

## 4. Discussion

CPP is a complex, often debilitating condition that is frequently accompanied by impairments in psychosocial and physical functioning, with limited treatment options available. CPP is typically addressed using invasive, peripherally focused treatments, such as surgical procedures (e.g., aimed at correcting suspected issues with anatomical structures in the urogenital or pelvic region) which do not target other dimensions of the pelvic pain experience [13, 14]. In fact, individuals with CPP often experience multiple other chronic pain conditions, and underlying mechanisms may be more centrally derived [7]. Given this, and the limited availability of interventions that target the multifactorial nature of CPP, understanding the mechanisms that may impact CPP is an important directive. Therefore, this study examined the relationships among COPCs, CS, and positive and negative affect in individuals with CPP.

Earlier work referencing “central sensitivity syndromes,” identified CS as a likely shared mechanism underlying what are now recognized as COPCs [37, 38]. Consistent with this framework, we found that a greater number of COPCs were associated with heightened CS, which aligns with extant literature supporting poorer outcomes in those with a greater number of COPCs [8, 36], as well as CS as a key contributor to the development of overlapping pain conditions [7, 9]. More recently, evidence suggests that the CS‐COPC relationship may also have genetic underpinnings, with one study showing that both the number of COPCs and reported CS symptoms share similar genetic influences [39]. Together, these findings reinforce the conceptualization of CS as a central biopsychosocial mechanism underlying COPCs.

Importantly, positive affect moderated the relationship between COPCs and CS. Specifically, a greater number of COPCs were associated with higher CS scores only among individuals reporting lower levels of positive affect, consistent with a low positive affect vulnerability pattern rather than a buffering effect. These findings suggest that lower positive affect is associated with greater susceptibility to CS symptoms among individuals with multiple COPCs. Supporting this, in patients with shoulder pain, lower positive affect and resilience were associated with greater widespread pain sensitivity—a marker of CS [16]. Similarly, positive affect has been shown to attenuate the impact of pain on negative affect in individuals with chronic back or knee pain [40]. Interestingly, negative affect did not moderate the COPC‐CS relationship in our sample, supporting the notion that negative and positive affect are distinct constructs rather than opposite ends of the emotion spectrum. As such, a reduction of positive affect may not equate to an increase in negative affect [23].

These findings underscore the role of positive affect in moderating the relationship between CS and overlapping pain conditions, highlighting it as a potentially modifiable factor that could be targeted in future interventions. Our findings are consistent with mounting evidence suggesting that lower positive affect, rather than an excess of negative affect, may be a key contributor to the emotional and physiological mechanisms linking depression and chronic pain [19, 40–43]. Interventions aimed at restoring positive affect by enhancing reward processing and positive emotional experiences, rather than solely focusing on reducing negative affect [44], may be promising in alleviating the burden of chronic pain but require further investigation in CPP samples. By addressing disruptions in reward sensitivity [44], such interventions could potentially facilitate more effective management of chronic pain and its associated emotional comorbidities.

A growing body of research supports the integration of positive activity interventions (PAIs) into chronic pain management, showing that these approaches can enhance positive affect and reduce pain‐related outcomes, such as catastrophizing and pain‐related interference [22, 45–47]. In individuals with chronic pain secondary to spinal cord injury, positive emotion induction via positive activity exercises led to greater pain reduction compared to a control condition, with treatment effects maintained over time [48]. Following a resilience‐promoting PAI, older adults with chronic low back pain reported improvements in pain intensity and pain‐related interference at post‐treatment and at the 3‐month follow‐up [49]. In addition, a recent Internet‐based intervention designed to cultivate positive affect in individuals with fibromyalgia demonstrated promising effects in increasing positive affect, while reducing negative affect and pain catastrophizing [50]. Other empirically supported treatments, such as behavioral activation, also aim to foster positive affect and have been shown to reduce depression [51]. Overall, these findings suggest that interventions focused on enhancing positive affect may have potential relevance for improving pain‐related outcomes in individuals with COPCs, particularly in light of the ongoing need for therapies that address central nervous system dysregulation in CPP [8, 52]. However, evidence specific to CPP populations remains limited, and the applicability of these approaches to CPP requires further investigation.

This study had some limitations that warrant acknowledgment. First, the use of a convenience sample recruited through online and panel‐based methods may have resulted in selection bias, and the cross‐sectional nature of the design limits our ability to determine the directionality of the associations. All data were self‐reported, preventing medical verification of the pain conditions and COPCs endorsed, which may have introduced misclassification bias, particularly given diagnostic heterogeneity across conditions. Future studies should confirm diagnoses through medical records or conduct a more formal diagnostic screening to assess symptomatology (e.g., Chronic Overlapping Pain Condition‐Screener [COPC‐S] [53]). Further, while count‐based approaches are a widely used method for capturing multimorbidity, representing COPCs as a simple count does not account for differences in condition severity or chronicity, nor does it capture specific patterns or combinations of conditions. Thus, this operationalization may not fully reflect the complexity of clinical burden associated with COPCs. Although the CSI is widely used to assess symptoms of CS, it remains an indirect measure that primarily captures somatic symptoms and psychological distress, which may overlap with comorbid disorders and complicate interpretation [54, 55]. Physiological and neurobiological markers of CS cannot be determined via a self‐report measure; however, the CSI provides a practical and cost‐effective method for assessing symptoms of CS, particularly in studies where objective approaches (e.g., quantitative sensory testing and neuroimaging) are not feasible [56]. Future research would benefit from incorporating objective measures to more directly capture the physiological processes underlying CS. Despite these limitations, the CSI remains a valuable tool for preliminary assessment and screening in clinical and research settings. Although the moderation model was statistically significant at low levels of PA, the effect size was small, signifying modest explanatory power at the individual level. As such, the clinical significance of the findings requires further clarification. Additionally, negative affect was highly correlated with CS though it was not directly associated with COPC burden, which could influence the interpretation of the models. Thus, caution is warranted in the interpretation of results, suggesting that future research is needed to clarify the role of affect in CS and COPCs. Lastly, participants were predominantly white, female, and highly educated, limiting the diversity of our sample and the ability to detect potential disparities related to race/ethnicity, sex/gender, and socioeconomic factors. This demographic pattern is partly consistent with the epidemiology of several COPCs, many of which occur more frequently in women [7], with some being female‐specific (e.g., vulvodynia and endometriosis).

Despite these limitations, this study had several strengths worth noting. This is the first known investigation of the independent impacts of positive and negative affect on the relationship between COPCs and CS in CPP. We recruited a large sample size, which included both males and females with CPP. Although our sample was heterogeneous in terms of reported pain type/duration, it is likely our recruitment methods allowed us to obtain a representative sample of individuals experiencing CPP, ultimately supporting greater generalizability of our findings. Continued investigation of factors that impact the mechanisms underlying CPP and COPCs is necessary. These future endeavors should aim to include greater sociodemographic diversity within recruited samples, as well as examination of additional positive, resilience‐promoting factors (e.g., optimism and pain acceptance) that may be related to CPP.

## 5. Conclusions

Our findings support the association between COPCs and CS in individuals with CPP, a relationship that was moderated by positive affect. Specifically, a greater number of COPCs were related to greater CS, but only in individuals with low positive affect. These results indicate that lower levels of positive affect are associated with a stronger relationship between COPCs and CS among individuals with CPP and comorbid chronic pain conditions. This highlights the importance of considering the potential benefits of enhancing positive affect when designing interventions for CPP. Future studies should continue to explore protective factors that confer healthy adaptation to the multidimensional nature of CPP, ultimately guiding the development of treatments that address central mechanisms contributing to pain and its broader impact.

## Funding

This work was supported by the National Institute of Arthritis and Musculoskeletal and Skin Diseases (NIAMS) under Award Number R01AR081835 (awarded to Emily J. Bartley) and the Eunice Kennedy Shriver National Institute of Child Health and Human Development (NICHD) under Award Number R21HD104957 (awarded to Emily J. Bartley and Meryl J. Alappattu) and K23HD104931 (awarded to Shreela Palit).

## Disclosure

The content is solely the responsibility of the authors and does not necessarily represent the official views of the National Institutes of Health.

## Ethics Statement

All procedures for this study were approved by the Institutional Review Board of the University of Florida.

## Consent

All participants provided informed consent before participating in the study.

## Conflicts of Interest

The authors declare no conflicts of interest.

## Data Availability

The data that support the findings of this study are available from the corresponding author upon reasonable request.
